# Combined Intra-Articular PN HPT™ and Hyaluronic Acid: Regeneration Medicine in Knee Osteoarthritis

**DOI:** 10.3390/jcm14093043

**Published:** 2025-04-28

**Authors:** Francesco Barcaro, Alessandro Cerino, Armando Francesco Cervini, Mario Gaffuri, Nikoleta Vaso, Mario Vela

**Affiliations:** 1 Istituti Clinici Città di Brescia, Via Bartolomeo Gualla 15, 25128 Brescia, Italy; 2 Cerino Center, Via Silvio Baratta 121, 84134 Salerno, Italy; 3 Casa di Cura Ospedale Privato Accreditato Salus, via Arianuova 38, 44121 Ferrara, Italy; 4 Orthopedic and Surgery, Moncucco Clinic, 6900 Lugano, Switzerland; 5 Unità di Medicina Interna, IRCCS Policlinico San Donato, Piazza Malan 1, 20097 San Donato Milanese, Milano, Italy; 6 ASL Napoli 1 Centro, S.da Comunale del Principe 13/a, 80145 Napoli, Italy; mariovela@alice.it

**Keywords:** knee osteoarthritis, hyaluronic acid, Lequesne index, numeric pain rating scale, PN HPT™, polynucleotides high purification technology, WOMAC

## Abstract

**Background/Objectives:** Natural-origin PN HPT™ (Polynucleotides High Purification Technology) protect and revitalize chondrocytes, synoviocytes, and cartilage with a regenerative medicine perspective following intra-articular injection. This six-month, open-label data collection aimed to validate the benefits documented in previous studies of a single intra-articular injection of a proprietary PN HPT™/HA-based medical device in improving both subjective and objective manifestations of knee osteoarthritis in real-life ambulatory patients of both genders with unilateral or bilateral knee osteoarthritis. **Methods:** Efficacy and safety assessments, conducted at baseline before a single PN HPT™/HA injection and after three and six months of follow-up, included the Lequesne index and the patient-assessed Numeric Pain Rating Scale (NPRS), which focuses on pain intensity, as primary endpoints. The Western Ontario and McMaster Universities Osteoarthritis Index (WOMAC) was a secondary endpoint. **Results:** After three and six months, the mean baseline Lequesne index score improved by 43.8% and 51.4%, respectively. Concurrently, the mean NPRS score improvements were 42.2% and 54.7%. Furthermore, 32% of investigators and 15.5% of treated patients deemed optimal the clinical outcomes with no clinical worsening. **Conclusions:** With some limitations due to the uncontrolled design and relying on subjective rating scales only, the study confirms all previous findings about the benefits of combining PN HPT™ and HA in the same medical device for intra-articular injection in knee osteoarthritis.

## 1. Introduction

Following the isolation of the non-sulfated glycosaminoglycan hyaluronic acid (HA) from the vitreous humor of the bovine eye (with hyaloid meaning vitreous) in 1934, veterinary medicine saw the first HA application in orthopedics, with the successful intra-articular HA injection in racehorses for relief of traumatic arthritis [1,2]. The 1970s saw the introduction of HA, physiologically synthesized by type B synoviocytes, for treating joint pain in humans [2]. HA, owing to its excellent viscoelastic properties at concentrations as low as 0.1%, along with high moisture retention, biocompatibility, and hygroscopic properties, acts as a potent lubricant, shock absorber, and joint structure stabilizer and helps maintain water balance and flow resistance regulation [3]. The foundation of the viscosupplementation concept developed by Balazs and Denlinger in their seminal 1993 paper was the idea of injecting exogenous HA intra-articularly into osteoarthritic diarthrodial joints such as the knee. The authors hypothesized that restoring synovial fluid rheology and possibly promoting the synthesis of higher molecular weight endogenous HA led to improved mobility, pain, and joint function [4].

Limiting HA viscosupplementation to a purely mechanical or rheological concept may be reductive. Chondrocytes, the unique resident cells of joint cartilage, act as mechanosensors and osmosensors and express the networks of proteins and glycoproteins that form the extracellular matrix of joint cartilage [2,3]. As osteoarthritis (OA) progresses, chondrocytes lose their ability to maintain cartilage homeostasis due to a decline in mitotic and synthetic activity [5]. Mainly mediated by interaction with the CD44 cell surface receptor (CD44 = cluster of differentiation protein 44) and CD44-mediated signaling, HA is likely to have a role in chondrocytes’ survival and apoptotic pathways [2,3]. At least in vitro, HA has a direct effect on chondrocyte survival by inhibiting nitric oxide-induced apoptosis and dedifferentiation, with a restored expression of aggrecan and Type-II collagen [6].

A therapeutic strategy for OA may be conceived that goes beyond a purely rheological and symptomatic approach by adopting a regenerative medicine perspective. Such a strategy could involve integrating the strong rheological properties and the beneficial, although modest, effects of HA on joint cells and cartilage with additional ingredients able to protect and revitalize chondrocytes and synoviocytes—all within the same medical device for intra-articular administration. As repeatedly demonstrated in single-agent studies, confirmed in combination with HA, and supported by preclinical evidence, intra-articular natural-origin, trout-derived polynucleotides, also known by the acronym PN HPT™ (Polynucleotides High Purification Technology), might help with such a regenerative medicine perspective [7,8,9,10,11]. PN HPT™, a no-prescription ingredient in CE-marked medical devices, might contribute further viscosupplementation owing to their high hydrophilic and viscoelastic properties and reduce pain more effectively and rapidly than HA. Moreover, PN HPT™ has well-documented reactivating properties on fibroblasts and chondrocytes [7,8,9,10,11]. In that sense, the PN HPT™/HA combination seems ideal. The primary contribution of PN HPT™ to the synergistic combination with HA is to provide nitrogen bases and precursors for nucleosides and nucleotides to fibroblasts, chondrocytes, and other mesenchymal cells, thus supporting the resilience and physiological functions of these cells in unfavorable environments such as osteoarthritis [7,8,9,10,11].

The illustrated prospective multicentric six-month multicenter study aimed to confirm the value and efficacy, repeatedly established in methodologically sound studies, of a single intra-articular injection of a proprietary PN HPT™/HA-based medical device in improving the subjective and objective manifestations of knee osteoarthritis [7,8,9,10,11].

## 2. Materials and Methods

Open-label, real-life clinical data were collected from unselected adult patients of both genders with unilateral or bilateral symptomatic knee osteoarthritis. Enrolled ambulatory patients should have displayed pain, muscle weakness, joint instability, brief morning stiffness, crepitus, and functional limitations for at least two months [12]. Only based on the investigator’s judgment, the enrollment strategy aimed to mirror the real-life, everyday orthopedic ambulatory practice. Due to the real-world confirmative nature of the investigation, there were no limiting inclusion and exclusion criteria. A known allergic diathesis was not a rigid exclusion criterion because of the negligible hypersensitivity potential of PN HPT™ as confirmed by previous studies in osteoarthritis of the knee and other sites.

After baseline evaluation, all patients received a single unilateral or bilateral 4 mL intra-articular injection of the proprietary PN HPT™/HA combination (CONDROTIDE HA medical device, Mastelli Srl, Sanremo, Italy). Efficacy and safety assessments, performed at baseline before a single PN HPT™/HA injection and after three and six months of follow-up, included the Lequesne index, Numeric Pain Rating Scale (NPRS) focused on pain intensity, the Western Ontario and McMaster Universities Osteoarthritis Index (WOMAC), overall safety, and treatment-emergent adverse events.

### 2.1. Primary Endpoints

The primary endpoints of the clinical data collection were the variations in disability and pain evaluated by the investigator using the Lequesne index (Table 1) and the Numeric Pain Rating Scale (NPRS) after three and six months (T1 and T2 visits, respectively), compared with the pre-injection baseline assessment (T0) [13,14].

The ten-question Lequesne algofunctional index includes three sections: “Pain or discomfort”(L1), “Maximum distance walked” (L2), and “Difficulties in performing activities of daily living” (L3) [13]. The Numeric Pain Rating Scale (NPRS) is an eleven-score (0 = no pain to 10 = extreme or worst possible pain) self-assessment scale for pain intensity, or other pain quality if requested, actual or experienced in the previous 24 h [14].

### 2.2. Secondary Endpoint

The secondary endpoint of the real-life clinical data collection was the variation, evaluated by the investigator after three and six months of follow-up, of the three-dimensional McMaster University OA (WOMAC) index—pain (five questions), stiffness (two questions), and physical function (seventeen questions) [15]. Three ordinal subscales with points (scores) ranging from 0 to 4 compose the Likert version of the WOMAC index. Each subscale is summated to maximum points of 20, 8, and 68, respectively.

Independently of each other, the investigators and the cohort patients assessed the overall clinical evolution of the symptomatic knee osteoarthritis as “Worsened”, “No change”, “Slightly improved”, “Improved”, and “Optimal results”.

### 2.3. Sample Size and Statistical Analysis

The sample size was estimated using the G*Power statistical program version 3.14 based on the worst-case hypothesis and considering two effect sizes. Based on available outcomes from published studies in knee osteoarthritis, the sample size calculation assumed a conservative 35% long-term improvement in the Lequesne index score after the PN HPT™ intra-articular injection. The minimum statistical power of detecting a significant two-tailed divergence compared with the expected Lequesne index score curve was set at 0.90 [statistical power: one less the ß-risk of false-negative type II errors]. Under those two assumptions, the estimated statistical power would have been greater than 0.91 with a study sample of 48 patients [16].

For both the primary and secondary efficacy endpoints, inferential statistics compared the evolution of the mean Lequesne index, NPRS, and WOMAC scores at baseline, T1, and T2 with the repeated-measure one-way ANOVA (within-subjects ANOVA, ANOVA for correlated groups) with time as the independent variable, the ideal statistical test to investigate changes in mean scores over three or more time points. The statistical analysis assessed whether the Lequesne index, NPRS, and WOMAC score curves diverged from the expected evolution (null hypothesis efficacy, +35%) [16]. All statistical tests were two-sided with a 5% significance level; statistical program: StatPlus release v7.

## 3. Results

### 3.1. Baseline Demographics and Dropouts

**Figure 1:**
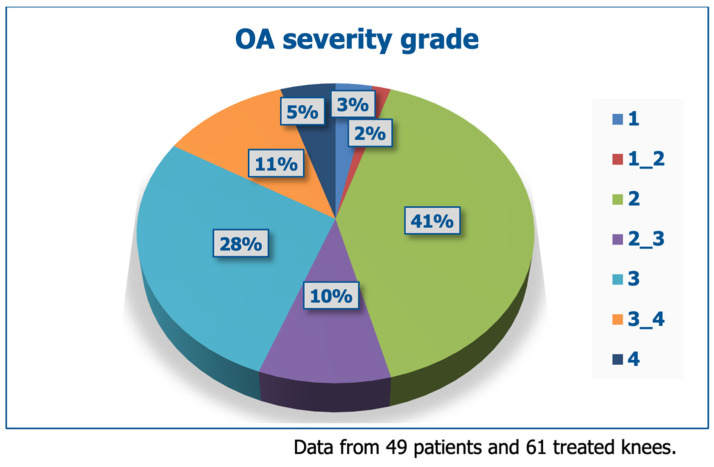
Clinical severity (OA grade) in the enrolled OA patients.

Fifty-four (54) patients of both genders were consecutively enrolled, with no specific selection criteria, to mirror the real-life ambulatory practice. Twelve (12) patients received bilateral treatment, for a total of sixty-six (66) joints treated with CONDROTIDE HA. Table 2 and Figure 1 illustrate the demographic data and baseline clinical severity of the treated patients; beyond pain, most patients experienced problems with motion.

All treated patients reported to their physicians at their program visits after three and six months to monitor the evolution of symptoms and knee function, with only occasional Lequesne, NPRS, and WOMAC scores missing, but no patient lost to follow-up.

### 3.2. Primary Endpoints

**Figure 2:**
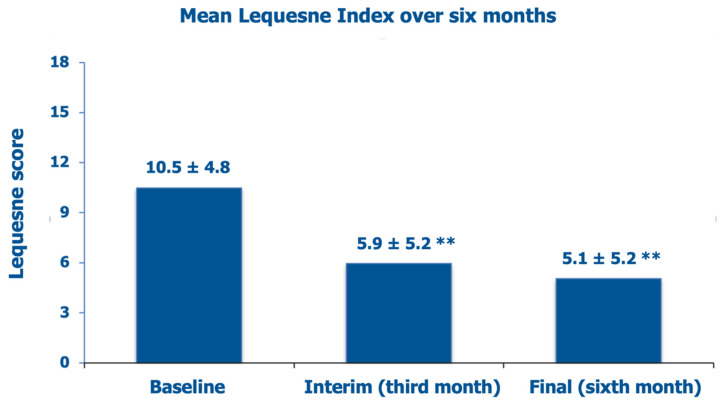
Evolution of the mean investigator-assessed Lequesne index scores over six months: baseline assessment before the PN HPT™/HA intra-articular injection, interim after three months, and final assessment (data on 65 knees); ** *p* < 0.01 vs. baseline.

The mean baseline Lequesne index score was 10.5 ± 4.8 (borderline score between “severe” and “very severe” symptoms and disability, with pretty frequent pain, stiffness, and difficulties in performing daily activities), improving to “moderate” clinical severity at the interim assessment after three months (−43.8% vs. baseline), with only occasional pain and stiffness but little trouble with performing daily activities (Figure 2). At the final visit after six months of follow-up, the Lequesne index had improved further within the “moderate” clinical severity class (−51.4% vs. baseline).

**Figure 3:**
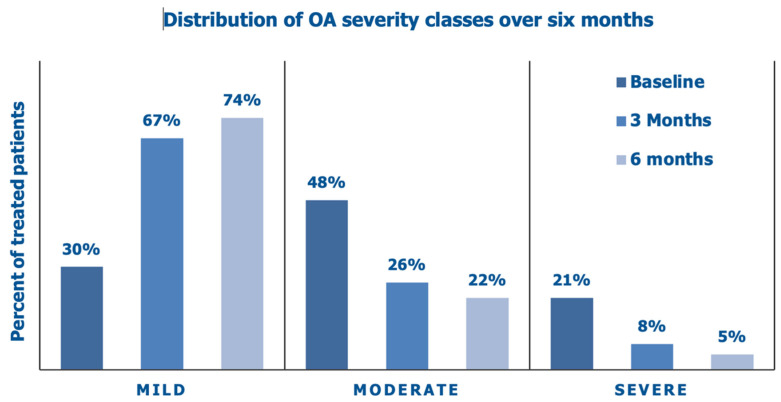
Distribution of clinical severity categories based on the Lequesne index over six months: baseline assessment before the PN HPT™/HA intra-articular injection, interim assessment after three months, and final assessment (data on 65 knees).

Figure 3 illustrates the improving distribution of the Lequesne severity categories over the six months of the prospective data collection and the steady decrease in OA patients with a moderate or high-grade Lequesne index.

**Figure 4:**
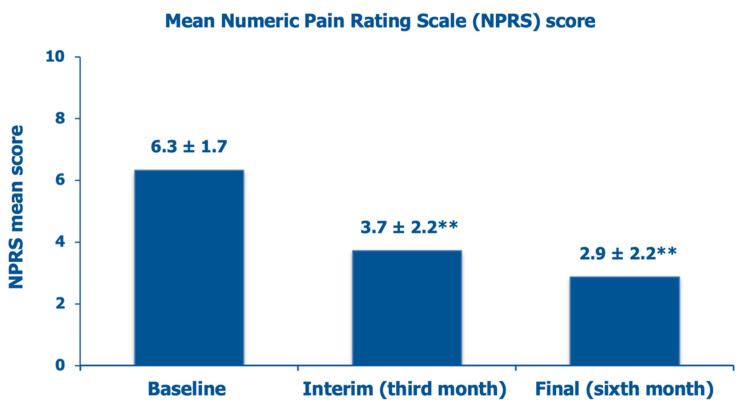
Evolution of the mean investigator-assessed NPRS scores over six months: baseline assessment before the PN HPT™/HA intra-articular injection, interim after three months, and final assessment (data on 65 knees); ** *p* < 0.01 vs. baseline.

The mean NPRS score, which was 6.3 ± 1.7 at baseline, a borderline score level between moderate (score range, 4–6) and severe (score range, 7–10) knee pain, decreased by 42.2% after three months and by 54.7% at the final follow-up assessment (Figure 4).

**Figure 5:**
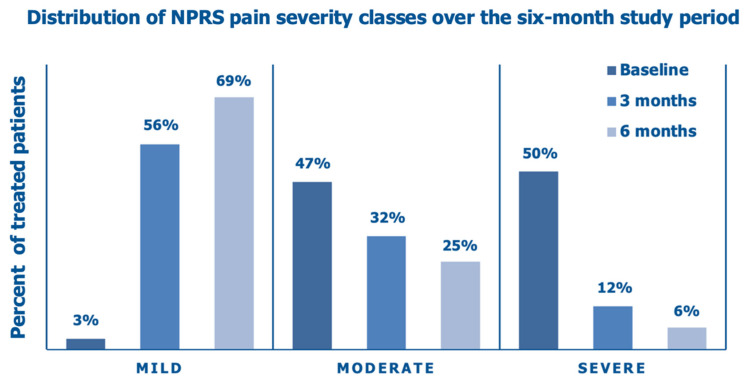
Distribution of PNRS pain severity classes over six months: baseline assessment before the PN HPT™/HA intra-articular injection, interim assessment after three months, and final assessment (data on 65 knees).

Figure 5 illustrates the improving distribution of the NPRS pain categories over the six months of the prospective data collection following the intra-articular PN HPT™/HA injection, with a steady shift from an overwhelming majority of the enrolled OA patients (97%) with moderate/severe baseline pain, stiffness, and interference with daily activities to a substantial majority of the same patients (69%) with mild pain, occasional discomfort, stiffness, and little interference with daily activities (NPRS score range, 0–3) at the end of the follow-up period.

### 3.3. Secondary Endpoint (WOMAC Score)

**Figure 6:**
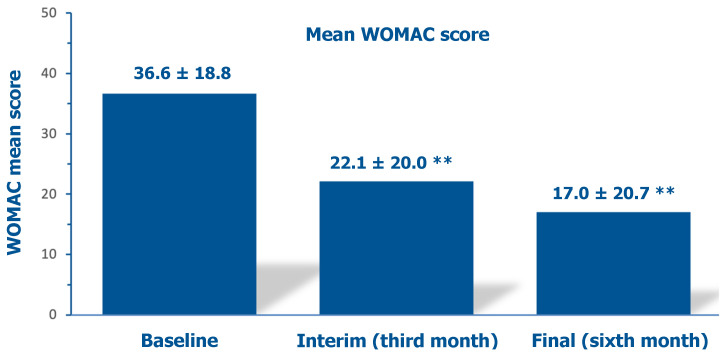
Evolution of the mean investigator-assessed total WOMAC scores over six months: baseline assessment before the PN HPT™/HA intra-articular injection, interim after three months, and final assessment (data on 65 knees); ** *p* < 0.01 vs. baseline.

**Figure 7:**
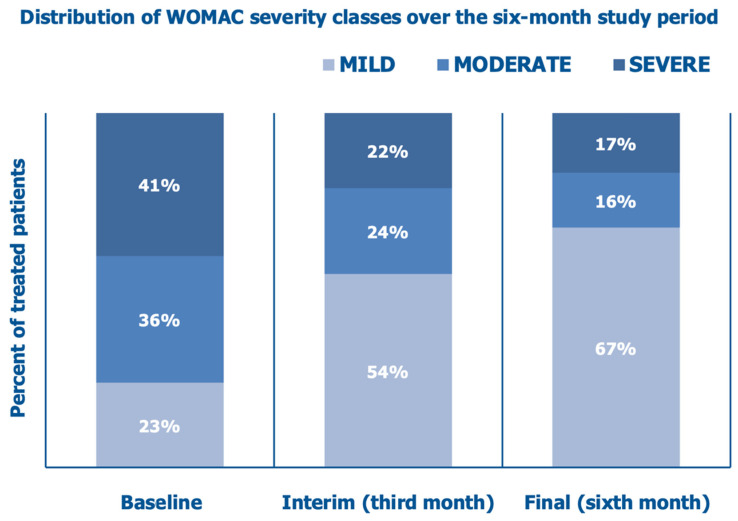
Distribution of WOMAC severity classes over six months: baseline assessment before the PN HPT™/HA intra-articular injection, interim assessment after three months, and final assessment (data on 64 knees).

The mean investigator-assessed total WOMAC score, 36.6 ± 18.8 at baseline, decreased by 39.6% after three months and by 53.6% at the final follow-up assessment (Figure 6). Figure 7 illustrates how the percent of cohort patients with severely or moderately symptomatic disease and handicaps in performing daily activities (WOMAC scores > 40 and between 21 and 40, respectively) progressively abated from 77% at baseline to 46% at the interim assessment and 33% at the final follow-up assessment after six months.

Table 3 illustrates how investigators and cohort patients independently assessed the overall symptomatic evolution of knee osteoarthritis. There was no side effect.

## 4. Discussion

The views on osteoarthritis have been evolving in recent years beyond the traditional perspective of a condition arising from the wearing and tearing of articular cartilage toward a vision of a chronic whole-joint disorder sparked by biochemical and cellular alterations in the synovial joint tissues. Inflammation, namely synovitis, is becoming key in explaining the frequent disconnect between radiographic findings and symptoms. New pieces of evidence are accumulating about the interactions, cross-talk, and activation states of joint-resident cells and inflammatory macrophages; the regulation of signaling pathways; and the interplay of proinflammatory and anti-inflammatory cytokines, chemokines, growth factors, and adipokines [17,18,19]. Examples of markers and signaling pathways assessable in serum, synovium, and histological samples and potentially useful as biomarkers of disease stage and progression are the Wnt/P-catenin, the TGF-ß/BMP, and the FGF pathways regulating cell proliferation, differentiation, and apoptosis; the NF-κB pathway involved in inflammation; the HIFs pathways regulating adaptation to hypoxia; and the intracellular regulators AMPK (AMP-activated protein kinase) for energy homeostasis, mechanistic Target Of Rapamycin (mTOR), and the RUNX2 gene controlling cartilage development and homeostasis [18].

The debate surrounding the actual value of HA in knee OA rages on, even in recent evidence-based guidelines supporting the likely benefits of combining HA with other intra-articular ingredients in the same medical device [20]. Beyond improving pain and mobility, many proposed strategies seek to restore the damaged cartilage or at least vicariate its function, enhance the congruence of joint surfaces, and prevent further deterioration of chondral morphology and function [21,22]. However, all proposed drugs, surgery, and other methods have never primarily focused on restoring the physiological microenvironment of the cartilage. Consequently, the biochemical and functional characteristics of the resulting fibrocartilage repair tissue inevitably differ from those of the physiological hyaline cartilage [23,24,25].

The disease burden associated with knee OA in developed countries suggests the benefits of aiming to restore the physiological microenvironment of cartilage as a primary therapeutic goal. In Italy, a survey conducted in the Chianti area in Tuscany among 1006 individuals over 65 years old highlighted that 22.4% lamented daily knee OA symptoms, often with severe disability and impressive societal costs, compounded by a further 7.2% lamenting both knee and hip OA symptoms [26].

Even as a single ingredient of medical devices for intra-articular OA treatment, natural-origin, highly purified PN HPT™ improves the fluid dynamics of synovial fluid with an intrinsic viscosupplementation efficacy. Furthermore, PN HPT™ tends to re-establish a proper physiological joint microenvironment, as evidenced in biopsies of atrophic cartilage after exposure to PN HPT™, leading to the production of Type-II collagen, aggrecan, and extracellular matrix (ECM) at levels comparable to normal controls [27]. Immunohistochemistry reveals that the in vitro ECM deposition by atrophic cartilage exposed to 1% or 2% PN HPT™ is abundant in Type-II collagen and similar to hyaline cartilage. Conversely, although new ECM production occurs following exposure to 1% or 2% HA, it is rich in Type-X collagen fibers and resembles fibrous scar cartilage [27]. Under environmental conditions that mimic the articular microenvironment of healthy hyaline cartilage, the optical density of fibers deposed after exposure to PN HPT™ matches the ground substance surrounding them, rendering those new fibers invisible within the ECM. Additionally, synoviocyte vitality is significantly higher after exposure to PN HPT™ compared with HA [27].

Considering all these factors, several previous studies have shown that the rationale for combining PN HPT™ and HA in the same medical device for intra-articular injection is compelling [7,8,9,10,11]. For instance, in a 2020 study, PN HPT™ appeared to be a reliable substitute for or complement to HA in knee and ankle OA. Enrolled patients with knee and ankle OA of mild to moderate severity showed substantial decreases in WOMAC and the 42-item Foot and Ankle Outcome Score (FAOS) mean scores after one month (−75.5% and −76.4%, respectively, compared with baseline), with substantial score decreases (−37.7% and −48.6%, respectively) in grades 3–4 patients who would typically be candidates for joint substitution [10]. Another 2021 study was a randomized, double-blind comparison of combined PN HPT™ and HA versus HA alone in gonarthritis management with a two-year follow-up. Patients of both groups experienced significant improvements in the KSS total score and the KSS pain item, but there was significantly more pain improvement in the PN HPT™ and HA group (2-point reduction) than in the HA alone group (1-point reduction) [9].

A recent study investigating the efficacy of PN HPT™/HA pericapsular injections to control the degenerative signs and related symptoms of mandibular condyle osteoarthritis (temporomandibular joint osteoarthritis) has further confirmed the rationale [28]. Microinvasive cosmetic plastic surgery has also reaped the benefits of the peculiar properties of PN HPT™ and the PN HPT™/HA combination [29,30].

Although the study can be a reasonably predictive photograph of real-life ambulatory practice in knee osteoarthritis, it has limitations: the non-selective data collection design, the lack of a control group, and relying solely on validated yet inherently subjective rating scales. An ongoing program of randomized clinical studies includes objective evaluations such as assessment of the joint range of motion and imaging-based cartilage thickness. Another of the study’s liabilities is the short follow-up of only six months, which does not allow for estimating a reasonable duration of effect. However, based on available experience from randomized studies, nine months to one year and possibly more may be a reasonable estimate [9].

A further liability originates from the study’s original purpose. When the authors designed their investigation, the PN HPT™ clinical research database in the management of knee osteoarthritis suffered from a paucity of data, mainly on safety compared with the more exhaustive data on efficacy. Therefore, the primary ambition of their six-month, open-label ambulatory data collection was to confirm the sparse safety evidence available until then. The authors thought an open-label single-arm design could be adequate and organizationally simple. Of course, the study design prevented all trustworthy conclusions about efficacy other than general support of previous evidence. Statistically, the possible data correlation bias arising from the inclusion of twelve patients treated bilaterally was considered to affect the analysis only negligibly and was ignored.

The PN HPT™ combination acts rapidly, with most symptomatic and mobility benefits, which seem impressive, reaped by the third week. Of course, the study can only pretend to confirm previous observations—what it does. The lack of a control group is a severe bias that prevents quantitative outcomes from being weighed against positive and negative controls.

## 5. Conclusions

The illustrated data collection confirms previous findings regarding the real-life benefits—beyond mere viscosupplementation—of combining PN HPT™ and HA in a single medical device for intra-articular injection in knee osteoarthritis. Even a single injection provides meaningful and long-term benefits for several months.

## Figures and Tables

**Table 1:** Descriptors of the Lequesne algofunctional index. Scoring: extremely severe (score greater than or equal to 14); very severe (score: 11 to 13); severe (score: 8 to 10); moderate (score: 5 to 7); mild involvement (score: 1 to 4) [13].

Pain or discomfort	Maximum distance walked (can walk with pain)
During sleep at night:	- Unlimited: 0
- None or insignificant: 0	- More than 1 km, but with some difficulty: 1
- Only when moving or in certain positions: 1	- Approximately 1 km (in about 15 min): 2
- Even without movement: 2	- From 500 to 900 m (approximately 8 to 15 min): 3
Morning stiffness or pain that decreases after getting up	- From 300 to 500 m: 4
- Only 1 min or less: 0	- From 100 to 300 m: 5
- More than 1 min, but less than 15 min: 1	- Less than 100 m: 6
- More than 15 min: 2	- With a cane or crutch: 1
After walking for 30 min: 0−1	- With two crutches or canes: 2
While walking	Daily activities/daily life *
- None: 0	- Can climb a flight of stairs: 0–2 *
- Only after walking some distance: 1	- Can go down a flight of stairs: 0–2 ′
- Soon after beginning to walk, and increases if continuing to walk: 2	- Squat or kneel: 0–2 *
- After beginning to walk, not increasing: 1	- Can walk on uneven ground: 0–2 *
When remaining seated for a long time (2 h) (only if hip): 0−1	
When rising from a chair without using the armrests (only if knee): 0−1	

′ Without difficulty: 0, With little difficulty: 0.5, With difficulty 1, With significant difficulty: 1.5, Unable: 2. * Applies only to knee

**Table 2:** Demographics of treated patients.

Age (years old, *)	Mean ± SEM, all patients	66.1 ± 12.8
	Range	34–90
Gender ^(^**^)^	Female	28 (53.8%)
	Male	24 (46.2%)
Weight (kg) ^(^***^)^	Mean ± SEM, all patients	74.0 ± 12.6
	Range	50–110
Height (cm) ^(^***^)^	Mean ± SEM, all patients	168.1 (8.6)
	Range	150–182
Which knee? ^(^****^)^	Right knee	23 (43.4%)
	Left knee	18 (34.0%)
	Both knees	12 (22.6%)

^(^*^)^ Data on 49 patients; ^(^**^)^ Data on 52 patients; ^(^***^)^ Data on 48 patients; ^(^****^)^ Data on 53 patients.

**Table 3:** Summary of final overall subjective clinical assessment by investigators and cohort patients (independent evaluation).

Evaluation	Investigator’s Assessment		Patient’s Self-Assessment	
	Patients	%	Patients	%
Worsened	0	0	0	0
No change	7	10.9	7	10.9
Slightly improved	16	25.1	20	31.3
Improved	20	31.2	27	42.2
Optimal results	21	32.8	10	15.6

Data on 64 treated knees in 52 patients.

## Data Availability

According to current regulations, the Corresponding Author has archived all datasets (clinical data (about ref. [27]: Mastelli internal clinical research report, R&D Code: DF02.1, Issued in Sanremo (Italy) on 17 January 2025. Available upon request) and iconographic documentation). All datasets are available upon reasonable request after being converted to an anonymous form.
